# Histological and Molecular Characterization of Grape Early Ripening Bud Mutant

**DOI:** 10.1155/2016/5620106

**Published:** 2016-08-17

**Authors:** Da-Long Guo, Yi-He Yu, Fei-Fei Xi, Yan-Yan Shi, Guo-Hai Zhang

**Affiliations:** College of Forestry, Henan University of Science and Technology, Luoyang, Henan 471003, China

## Abstract

An early ripening bud mutant was analyzed based on the histological, SSR, and methylation-sensitive amplified polymorphism (MSAP) analysis and a layer-specific approach was used to investigate the differentiation between the bud mutant and its parent. The results showed that the thickness of leaf spongy tissue of mutant (MT) is larger than that of wild type (WT) and the differences are significant. The mean size of cell layer L2 was increased in the mutant and the difference is significant. The genetic background of bud mutant revealed by SSR analysis is highly uniform to its parent; just the variations from VVS2 SSR marker were detected in MT. The total methylation ratio of MT is lower than that of the corresponding WT. The outside methylation ratio in MT is much less than that in WT; the average inner methylation ratio in MT is larger than that in WT. The early ripening bud mutant has certain proportion demethylation in cell layer L2. All the results suggested that cell layer L2 of the early ripening bud mutant has changed from the WT. This study provided the basis for a better understanding of the characteristic features of the early ripening bud mutant in grape.

## 1. Introduction

Grape (*Vitis vinifera* L.) is one of the most widely cultivated fruit trees in the world, which have been cultivated for thousands of years for fresh fruit, dried fruit, and wine production. There are thousands of grape varieties in the world. Many of these varieties have been derived from crosses among or between species to produce new cultivars. The most important group is from crosses between* V. vinifera* and* V. labrusca*, such as “Kyoho” which is developed to produce a large berry [1].

“Kyoho” is one of the most important grown varieties in the world today. It was introduced to China in 1959 from Japan. It has many eminent advantages, large berries, high production, and adaptation to high temperature, rainy, and wet environments [2]. An early ripening bud mutant of “Kyoho,” “Fengzao,” was recently presented [2]. It matures in early July in Henan Province, China, and nearly one month earlier than “Kyoho.” All of its traits are similar to those of “Kyoho” except the ripening date. The pehontypic and physiological differences between the bud mutant and its parent have been investigated in detail [3, 4].

Bud mutants arising from somatic variants are important genetic materials for cultivar improvement in grape [5]. Any new desirable trait in a given bud mutant could be fixed in grape by vegetative propagation such as grafting. Bud mutants have been widely exploited by vine growers to develop new cultivars of wine grapes and table grapes [5].

Variant traits of bud mutants in grape may include color or flavor, date of ripening, and canopy growth, size, and cluster architecture [5]. Spontaneous mutations in* Vitis* have been studied by some researchers [6–9]. These mutations could be present in the entire meristem or only a portion (chimeras) [6]. In grape, the shoot apical meristem (SAM) is considered to be composed of only two (L1 and L2) genetically distinct cell layers [5, 6, 10]. In some cases, bud mutants affect only one-cell layer in grape, resulting in periclinal chimeras [6] which is a specific structure type of genetic mosaic; that is, the genetic makeup of one-cell layers of the apical meristem is distinct from the others and develops independently from the adjacent layers [5, 11].

In few cases, the small mutations that lead to bud mutant are observable within the noncoding DNA associated with SSR markers in grape [12]. Three- and four-allele genotypes, indicating chimeric structures, have been detected using SSR markers in some varieties [6, 8, 9, 13].

The molecular mechanisms of bud mutants have been hypothesized as gene mutation, transposon activity, and DNA methylation or various combinations of these effects [6, 14]. DNA methylation has been considered a key regulator of gene expression. The DNA of most eukaryotic organisms contains 5-methylcytosine (mC) residues, which is involved in the regulation of gene expression during various developmental processes [15]. Several researchers had reported that methylation patterns vary among the bud mutant and the parent line [16–18]. Recent studies have shown that DNA methylation plays important roles in regulating fruit development and ripening [19].

Reyna-López et al. [20] developed the methylation-sensitive amplified polymorphism (MSAP) method based on the different methylation-sensitive restriction enzymes and modification of the amplified fragment length polymorphism (AFLP) technique. Due to its advantages, such as simple operation, the high number of available polymorphisms, and convenient primer design, the MSAP technique has been used widely to analyze DNA methylation changes in plants [21, 22].

The aim of this study was to investigate the differentiation between the early ripening bud mutant and its parent lines based on the histological, SSR, and methylation-sensitive amplified polymorphism (MSAP) analysis using a layer-specific approach.

## 2. Materials and Method

### 2.1. Plant Material and Genomic DNA Extraction

An early ripening bud mutant (“Fengzao”) and its parent (“Kyoho”) were studied. The samples were collected from the experimental vineyard of Henan University of Science and Technology located in the county of Yanshi, Luoyang, China (34.41°N, 112.46°E). The mean annual temperature is 14.2°C. During the period of early April and late September, the average day length is 13.8 h. Phenological traits were investigated in 2013 according to Coombe [23] and Rustioni et al. [24]. The layer-specific approach was performed as described by Vezzulli et al. [8] based on the theory that leaf and berry skin are derived from L1 + L2 layer and berry flesh and root only from L2 layer [8, 9]. Therefore, genomic DNA of each cultivar from the same vine was extracted three times from 300–500 mg of young leaf, berry skin, berry flesh, and root, respectively, using the modified CTAB extraction protocol [25].

### 2.2. Histological Analysis

Leaf properties and anatomical measurements were conducted according to the method of Cai et al. [26]. Small pieces from the middle leaves were cut and fixed in FAA (formalin/glacial acetic acid/50% ethanol, V/V/V, 5/5/90) for 24 h, then dehydrated by gradient ethanol, cleared in xylene, and at last embedded in paraffin. After that, 8 *μ*m thick sections were cut using a Leica RM2265 microtome (Leica Biosystems, Germany) and then mounted on glass slides. Leaf thickness, epidermis, palisade tissue, and spongy tissue were measured with a Nikon Eclipse E800 light microscope (Nikon, Melville, NY, USA) at 400x magnification.

The measurements of the guard cell size and the stomata density were according to the method of Xu and Zhou [27]. An epidermal impression from the abaxial epidermis of the leaf was prepared by coating the leaf surface with nail varnish and then peeling off the dried layer of nail varnish by using sellotape and sticking this onto a slide. Guard cell lengths (*μ*m) were measured to the nearest micrometer viewed at 40x magnification. Three leaves per plant and three fields per leaf were used to determine stomata density. Nine measurements were taken for guard cell length and width for each field.

The measurement of SAM is based on the method of Jouannic et al. [28]. The shoot tip of the new growing twig was selected and then examined using the routine methods of paraffin section as above.

All the above histological analyses were repeated 3 times, respectively. Statistical significance was determined by using* t*-test for comparisons. Significance levels were compared at *p* < 0.05 and the analysis was performed by SPSS 20.0 software.

### 2.3. Single Sequence Repeat (SSR) Analysis

Molecular characterization was carried out using 14 SSR markers for each sample. The SSR loci and annealing temperatures used for polymerase chain reaction (PCR) analysis are listed in Table 1. Primer sequences were obtained from Vezzulli et al. [8]. PCRs were carried out in a final volume of 20 *μ*L, containing 30 ng of DNA template, 1x PCR buffer, 1.5 mM MgCl_2_, 0.3 mM of each dNTP, 0.4 mM forward and reverse primer, and 1.0 U of* Taq* DNA polymerase (TaKaRa, Dalian, China). Amplification was carried out using the following cycling profile: initial denaturation at 94°C for 5 min, followed by 35 cycles of 94°C for 1 min, 54–68°C for 1 min (see Table 1), and 72°C for 1.5 min and a final extension step at 72°C for 8 min. The PCR products were separated on 6% (w/v) polyacrylamide gels and visualized by silver staining.

### 2.4. Methylation-Sensitive Amplification Polymorphism Assay

The MSAP protocol was modified from Reyna-López et al. [20]. The genomic DNA (200 ng) was double-digested with* Hpa* II/*EcoR* I or* Msp* I/*EcoR* I (MBI, USA) at 37°C for 2 h according to the manufacturer's instructions. The reactions were terminated by incubating the samples at 65°C for 20 min. The digested DNA fragments were ligated to the double-stranded* EcoR* I adapter and the* Hpa* II/*Msp* I adapter simultaneously [20]. The ligated DNA was diluted to 1 : 5 and then preamplified using* EcoR* I and* Msp* I or* Hpa* II primer with one selective nucleotide at the 3′ end each. The adapters, preamplification primers, and selective amplification primers are the same as Ocaña et al. [29] used.

A preamplification reaction was carried out in a total volume of 20 *μ*L, containing 0.4 mM dNTPs, 1x buffer, 1.0 U/*μ*L* Taq* polymerase (TaKaRa, Dalian, China), 0.5 *μ*M E01-primer, and HM0-primer. The preamplification PCR reaction protocol consisted of 25 cycles at 94°C for 30 s, 56°C for 30 s, and 72°C for 1 min with a final extension at 72°C for 10 min.

The preamplification products were diluted 1 : 10 with sterilized ultrapure water for further selective amplification using different combinations of* EcoR* I and* Msp* I or* Hpa* II primer each with three selective nucleotides at the 5′ and 3′ end, respectively. Selective amplification was conducted in a volume of 20 *μ*L, containing 0.4 mM dNTPs, 1x buffer, 1.0 U* Taq* polymerase, 0.5 *μ*M* EcoR* I selective amplification primers, and* Hpa* II/*Msp* I selective amplification primers. Selective amplifications were performed using 65°C as the initial annealing temperature for the first cycle and for the subsequent 11 cycles the annealing temperature was successively reduced by 0.7°C. Twenty-three cycles were run at 56°C annealing temperature. In total, 14 selective primer combinations were employed according to Ocaña et al. [29]. The samples were then resolved by electrophoresis on a 6% denaturing polyarylamide gel (PAGE, 6% polyacrylamide). The polyarylamide gel was stained according to the silver staining method and photographed.

### 2.5. Profiling Scoring and Data Analysis

In PAGE profiles the bands present in both* EcoR* I/*Hpa* II and* EcoR* I/*Msp* I lane were considered as type I (nonmethylated), in* EcoR* I/*Msp* I lanes, but not in* EcoR* I/*Hpa* II as type II (full- or hemimethylated internal cytosine), in* EcoR* I/*Hpa* II, but not in* EcoR* I/*Msp* I lane as type III (hemimethylation of external cytosine), and absent in both the lanes as type IV (uninformative). The absence of bands in both the* Msp* I and* Hpa* II lanes could be caused by either restriction target absence or hypermethylation [22, 30].

## 3. Results and Discussion

### 3.1. Comparison of Phenological Traits

**Figure 1:**
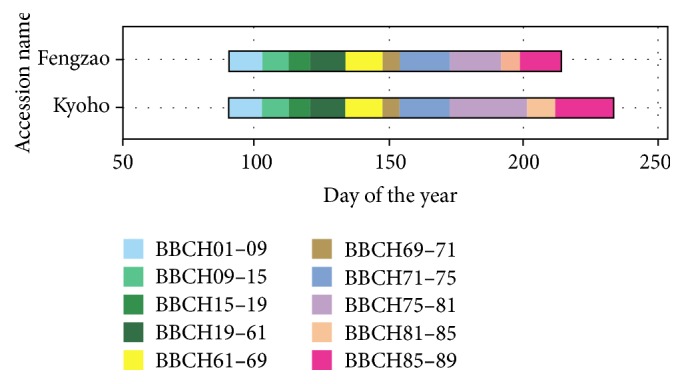
Phenological variance between “Fengzao” and “Kyoho.” The color changes represent different growing stages according to Rustioni et al. [24].

Phenological traits were investigated in detail in 2013 from the period of the beginning of bud swellings to the period of berries ripe for harvest. The phenological variability was showed in Figure 1. The specific growing stages were showed in different colors. “Fengzao” and “Kyoho” have the exact synchronous developing process before BBCH (Bundessortenamt and Chemical Industry) phenophases 75 (berries pea-sized). After the BBCH 75, the phenological difference between “Fengzao” and “Kyoho” is distinct. The period from BBCH 15 to 81 of “Fengzao” is 12 days shorter than that of “Kyoho.” The ripening advance of “Fengzao” is related to an advance of the phenological phases (veraison) and shortening of the ripening phase.

### 3.2. Histological Analysis

Both the length and width of the guard cell in the early ripening mutant (MT) are larger than its parent line (WT), respectively. But the differences are not significant between them with* p* values of 0.05 (Table 2). There are also not significant differences in the thickness of upper epidermis and lower epidermis of the leaf between WT and MT. The thickness of leaf spongy tissue of MT is larger than that of WT and the differences are significant (Table 2).

There are two layers in the SAM of grape, L1 and L2. The results of the measurement of the L1 and L2 layer showed that the length and width of L1 and L2 in MT are larger than those of the parent line (Table 3). The difference in L1 is not significant. However, significant difference is observed in L2 (Table 3).

Shoot apical meristems (SAMs) are small groups of dividing cells that initiate all of the aerial parts of the plant [31]. In dicots, three layers can be distinguished, L1, L2, and L3. Leaves in most eudicot species are composed of derivatives from the epidermal layer (L1), the subepidermal layer (L2), and corpus (L3) [32]. It is known that L1 gives rise to the leaf epidermis including the guard cells that surround stomata. L2 generates the palisade parenchyma and the lower spongy parenchyma as well as all of the spongy parenchyma of the leaf margin [33].

There is some difference for L1 layer (epidermal, guard cells) between WT and MT, but it is not significant. It demonstrated that there are no distinct differences in L1 layer between WT and MT. The thickness of palisade parenchyma and spongy parenchyma (L2 layer) in MT is larger than that of WT, and the difference of spongy parenchyma is significant, which suggested there are distinct differences in L2 layer between WT and MT. In addition, variations in the size of L1 and L2 cell layer were observed between the meristems of WT and MTs. The L2 meristems in MT are wider and longer than the WT meristems. The mean size in the L2 layer was increased in mutant when compared with the wild type and it is significant.

### 3.3. SSR Analysis

SSR marker has been employed to detect polymorphisms at the clonal level in Pinot [13], Chardonnay [34], and Tempranillo [35], and so forth. In order to determine the differences of the genetic constitution of WT and MT, 14 SSR markers were used to discriminate them.

For “Kyoho” and “Fengzao,” out of the 14 SSR primers used for the 8 DNAs, only 1 (VVS2) gave different alleles. Three alleles were detected in leaves, root, berry skin, and berry flesh of “Kyoho” but just 2 alleles in the corresponding parts of “Fengzao.” VVS2 has been detected as triallelic profile in many studies as the most frequent difference detected alleles in the different clones of the same cultivar [6, 8, 13]. The apical meristem of the grape is made up of two-cell layers [10]. In the leaf, berry skin, berry flesh, and root tissue of “Fengzao,” the SSR marker VVS2 all revealed the two alleles. But for “Kyoho,” VVS2 all showed a variant allele besides the two alleles in these tissues. This allele is likely to have mutated in “Fengzao.”

Polymorphisms identified by SSR markers have shown the presence of triallelic loci, referred to in grape as chimeras [12, 13] produced by mutations in cells of the meristem layers L1 or L2 [10]. Because the leaf and berry skin tissues are derived from cell layers L1 and L2, the berry flesh and root tissues are derived only from cell layer L2; the lost allele in “Fengzao” suggested there should be some difference in cell layer L2 between “Kyoho” and “Fengzao” but it is not sure whether there is difference in cell layer L1 or not between them because the leaf and berry skin (L1 + L2) of “Kyoho” are also different from those of “Fengzao.”

This study confirmed that the genetic background of bud mutants is highly uniform with its parent and showed the VVS2 SSR mutation in “Fengzao.” The ampelographic differences observed in MT and WT probably reflect epigenetic differences.

### 3.4. MSAP Analysis

MSAP usually uses two isoschizomers systems:* EcoR* I/*Msp* I and* EcoR* I/*Hpa* II.* EcoR* I is insensitive to DNA methylation and cuts 5′-GAATTC-3′ sites. Both of* Hpa* II and* Msp* I could recognize and cleave the same 5′-CCGG-3′ sites but differ in their sensitivity to the methylation state of cytosine.* Hpa* II cuts when only the external cytosine is home- (single strand) methylated, and* Msp* I cuts when only the internal cytosine is hemi- or fully (double strand) methylated [22, 36]. Owing to the differential sensitivity of* Msp* I and* Hpa* II to the methylation state at 5′-CCGG-3′ sites, it was possible to define whether a demethylation or methylation event had occurred in a single MSAP locus between the bud mutant and its parent [37].

Epigenetic variation could take place in a faster way compared to genetic variation [29]. In grape, Schellenbaum et al. [17] have used MSAP to study somaclonal variation in “Syrah” and “Chardonnay” cultivars allowing the identification of methylation alterations and possible methylation hotspots.

In order to compare the different extent of methylation between the WT and MT, MSAP was employed in this study and the results are shown in Table 4. A total of 536 and 550 different MSAP fragments were scored in “Kyoho” and “Fengzao” after restriction with* EcoR* I/*Msp* I and with* EcoR* I/*Hpa* II, respectively (Table 4). In general, divergences of occurrence of methylation events between them were evident. The total methylation ratio of MT is lower than that of the corresponding WT (20.5% < 26.3%) and the difference is significant. So, it means that the main difference between MT and WT is due to the demethylation.

The proportion of type III fragments (31, 5.6%) in “Fengzao” is far below that of “Kyoho” (70, 13.1%), while the proportion of type II fragments (82, 14.9%) in “Fengzao” is higher than that of “Kyoho” (Table 4). But the total methylation ratio in “Fengzao” is still lower than that of “Kyoho.” It suggested that there is more demethylation that occurred at the external cytosine than methylation that occurred at the internal cytosine in “Fengzao” when it mutated from “Kyoho.”

In order to characterize the methylation differentiation of L1 + L2 and L2 between WT and MT, the data were further analyzed for methylation rate in L1 + L2 and L2-derived tissues, respectively (Table 5). The results showed that the total methylation ratio in both L1 + L2 and L2-derived tissues in MT is lower than that in WT. For only L2, the outside methylation ratio in both MT is much less than that in WT; the average inner methylation ratio in MT is larger than that in WT. It means demethylation occurred at the external cytosine and methylation at the internal cytosine happened in cell layer L2 of MT when they mutated. In other words, the cell layer L2 of the early ripening bud mutant has changed from the original parent line. For L1 + L2, the situation is complicated. It could not conclude whether it has changed or not because the up and down of the methylation ratio are not fixed. Our approach did not rely on pure L1-derived tissues, which are difficult to obtain or isolate, and this is the reason why it was not possible to verify if the mutation affected L1 as well as L2 cells.

There is a higher level of DNA methylation detected in the promoter region of MdMYB1 in the apple cultivar “Ralls” than in its blushed sport [16]. Similarly, the research in European pear “Max Red Bartlett” has also shown that its red-color-loss mutation was also due to DNA methylation in the PyMYB10 promoter [38]. The study in the tomato showed that the methylation of some genes related to fruit ripening (*CNR*,* NOR*, and* RIN*) restrained their expression and then inhibited the ripening of the fruit, while the demethylation of these genes will activate the ripening process [39]. All of these studies showed that the methylation state of the corresponding genes has the important influences on the trait formation of the bud mutants. This study has showed that the early ripening bud mutants have certain proportion demethylation in cell layer L2 that occurred which may be due to the demethylation of the ripening related genes.

## 4. Conclusions

Combined with the histological, SSR, and MSAP analysis, there is no doubt that the cell layer L2 of the early ripening bud mutant has changed from the WT. This study has involved more than one technique to generate enough information to explore the genetic differentiation between the early ripening bud mutant, “Fengzao,” and its parent line, “Kyoho.” MSAP and SSR markers were found to be efficient technologies that can identify the variants and explain the underlying mechanism behind the resulting variations.

## Figures and Tables

**Table 1:** 14 SSR primer sequences used in this study.

Primers	Sequence	Annealing temperature
VVS2	F: CAGCCCGTAAATGTATCCATC R: AAATTCAAAATTCTAATTCAACTGG	58.0 52.1
VVMD5	F: CTAGAGCTCGCCAATCCAA R: TATACCAAAAATCATATTCCTAAA	57.8 50.0
VVMD7	F: AGAGTTGCGGAGAACAGGAT R: CGAACCTTCACACGCTTGAT	57.8 57.8
VVMD27	F: GTACCAGATCTGAATACATCCGTAAGT R: ACGGGTATAGAGCAAACGGTGT	60.5 60.1
VRZAG62	F: GGTGAAATGGGCACCGAACACACGC R: CCATGTCTCTCCTCAGCTTCTCAGC	66.9 65.3
VRZAG79	F: AGATTGTGGAGGAGGGAACAAACCG R: TGCCCCCATTTTCAAACTCCCTTCC	63.6 63.6
VVMD25	F: TTCCGTTAAAGCAAAAGAAAAAGG R: TTGGATTTGAAATTTATTGAGGGG	55.1 55.1
VVMD28	F: AACAATTCAATGAAAAGAGAGAGAGAGA R: TCATCAATTTCGTATCTCTATTTGCTG	57.6 57.4
VVMD32	F: TATGATTTTTTAGGGGGGTGAGG R: GGAAAGATGGGATGACTCGC	58.4 59.8
VMC5G6-1	F: TTCTAAGACAGAATTGCTTGGC R: TTATCTGTAGCTTTCACACCCC	56.3 58.2
VMC8F10	F: TATGAAAGATGAATGGCTGCTC R: AAGGGTGCTTGAAGGTTTATGT	56.3 56.3
VMC1E8	F: CAGCGAGCTCTTGATTTATTGT R: GATCATAGCTTCAACGGCTTTT	56.3 56.3
VMC3B12	F: ATAAGGCAGGTTGATTACAGGA R: CATCACAGGTTGATTCGACACT	56.3 58.2
VMC3C9	F: ATAAAATGGAATTAAGGGGGGA R: CAAACGCTAGATACCATGGAGA	54.5 58.2

**Table 2:** Size of guard cell and stomata density among experimental varieties.

Cultivars	Length of guard cell (*µ*m)	Width of guard cell (*µ*m)	Upper epidermis thickness (*µ*m)	Lower epidermis thickness (*µ*m)	Palisade parenchyma thickness (*µ*m)	Spongy parenchyma thickness (*µ*m)
Kyoho	29.79 ± 3.69	19.64 ± 3.39	14.55 ± 1.94	10.72 ± 1.87	40.22 ± 3.73	59.10 ± 4.16
Fengzao	30.17 ± 3.87	19.86 ± 3.24	14.45 ± 1.85	11.28 ± 1.53	44.99 ± 3.28	64.62 ± 5.63^*∗*^

“*∗*” indicates the significance of differences (*p* < 0.05).

**Table 3:** Size of stem apex cell between experimental varieties.

Cultivars	L1		L2	
	Length/*µ*m	Width/*µ*m	Length/*µ*m	Width/*µ*m
Kyoho	7.19 ± 2.27	5.85 ± 2.11	7.85 ± 3.93	6.29 ± 3.06
Fengzao	7.81 ± 2.30	6.45 ± 1.68	11.64 ± 2.56^*∗*^	9.38 ± 1.84^*∗*^

“*∗*” indicates the significance of differences (*p* < 0.05).

**Table 4:** Results of MSAP analysis of genome DNA methylation of grape varieties.

Cultivars	Type of bands			Total bands	Inner methylation ratio	Outside methylation ratio	Ratio of methylation
	I	II	III				
Kyoho	395	71	70	536	13.2%	13.1%^*∗*^	26.3%^*∗*^
Fengzao	437	82	31	550	14.9%	5.6%	20.5%

Note: total bands = I + II + III; the ratio of outside methylation = II/(I + II + III); the ratio of inner methylation = II/(I + II + III); ratio of methylated loci = II + III/(I + II + III).

“*∗*” indicates the significance of differences (*p* < 0.05).

**Table 5:** Analysis of methylation levels in grapes L2 with 14 MSAP primer combinations.

Cultivars	The outside methylation ratio		The inner methylation ratio		Total methylation ratio	
	L1 + L2	L2	L1 + L2	L2	L1 + L2	L2
Kyoho	9.4% ~13.0%	11.2% ~19.8%	12.3% ~19.5%	6.9% ~12.8%	25.3% ~28.9%	24.0% ~26.7%
Fengzao	5.7% ~6.2%	3.6% ~6.7%	15.2% ~17.1%	13.3% ~13.5%	20.9% ~23.3%	17.1% ~20.0%
